# Psychometric properties of the short mood and feelings scale among Chinese adolescents using item response theory

**DOI:** 10.3389/fpsyt.2025.1561728

**Published:** 2025-06-13

**Authors:** Fang Wang, Xuliang Gao

**Affiliations:** School of Psychology, Guizhou Normal University, Guiyang, China

**Keywords:** short mood and feelings questionnaire, item response theory, psychometric properties, differential item functioning, adolescents

## Abstract

**Background:**

Depression is a common mental health condition that can manifest at various stages of life, including the early stages such as childhood and adolescence. In particular, adolescence is a critical period where depression can present with numerous significant and severe symptoms, such as persistent sadness, behavioral changes, and difficulties in academic performance and social interactions. These symptoms, if left untreated, can have long-lasting effects and may recur in adulthood. Early identification and monitoring of depression are therefore essential to ensure timely intervention.

The Short Mood and Feelings Questionnaire (SMFQ) is a widely used tool for measuring depression symptoms in adolescents. This study aimed to assess the SMFQ using Item Response Theory (IRT) in adolescents and determine optimal cutoff points for a revised version.

**Methods:**

Using IRT and the Graded Response Model (GRM), we evaluated the SMFQ in 906 Chinese adolescents (average age 15 years). Items 1, 3, 4, and 6 were removed, resulting in the SMFQ-9. Reliability and validity were assessed using Cronbach’s alpha, and Receiver Operating Characteristic (ROC) analysis was conducted to determine cutoff points.

**Results:**

We validated the reliability and validity of the SMFQ-9, with the structure showing a Cronbach’s alpha as high as 0.86. It achieved significant correlations with three criterion questionnaires, and the correlation between SMFQ-9 and full version SMFQ reached 0.975. ROC analysis established an optimal cutoff value of 4.5, with an AUC of 0.985.

**Conclusions:**

The SMFQ-9 retains the robustness of the original SMFQ, improves efficiency, reduces respondent burden, and is a reliable tool for assessing mood in adolescents in clinical and research settings.

## Introduction

Adolescence is an important stage of rapid physical and mental development, with multiple developmental issues, and globally adolescents are at high risk for mental health problems. Mental health problems among adolescents not only lead to personal suffering and family burdens, but also have potential negative impacts on social development. Depression is among the most prevalent mental health issues in adolescents, serving as a significant risk factor for suicide and adversely impacting cognitive, social, and academic development. In a large-scale survey in China, more than 30,000 primary and secondary school students aged 10–16 years were assessed for mental health, and the results showed that about 14.8 per cent of adolescents were at varying degrees of risk for depression, with 4.0 per cent at risk for severe depression and 10.8 per cent at risk for mild depression (1).

Although depressive disorders are common in children and adolescents, many affected individuals do not seek or receive psychiatric evaluation or treatment. The lack of appropriate intervention can have serious consequences. Untreated depression can lead to psychological sequelae, making these young individuals more susceptible to recurrent depressive episodes, impaired occupational functioning, and reduced life satisfaction (2). Accurate identification of adolescent depression is crucial because it can prevent a range of adverse outcomes. Depression in children and adolescents is associated with poor academic performance, difficulties in securing and maintaining employment, an increased risk of self-harm, and a higher likelihood of experiencing depression in adulthood (3).

Currently, several screening questionnaires have been developed to detect depression and depressive symptoms in children and adolescents. These tools are essential for early diagnosis and intervention. Notable among them are the Children’s Depression Rating Scale—Revised (CDRS‐R) (4), which is widely used for clinical assessments; the Reynolds Adolescent Depression Scale—Second Edition (RADS‐2) (5), designed to evaluate the severity of depressive symptoms in adolescents; the Moods and Feelings Questionnaire (MFQ) (6), which helps in identifying mood disorders in younger populations; and the Short Moods and Feelings Questionnaire (SMFQ) (7), a brief and efficient tool for screening depressive symptoms. These instruments are invaluable for clinicians and researchers aiming to understand and address depression in young populations, ultimately aiding in the prevention of long-term negative outcomes associated with untreated adolescent depression. The Mood and Feelings Questionnaire (MFQ) is a depression screening tool consisting of 33 questions for children and young people and is the recommended screening tool for depression in children and young people (8). The Short Mood and Feeling Questionnaire (SMFQ) has 13 items selected from the MFQ that cover depressive symptoms in DSM and ICD. Both MFQ and SMFQ has been validated in clinical and non-clinical samples (9–12). The SMFQ is more attractive than the MFQ because it is less time-consuming for initial screening for clinical disease and measurement of clinical change in large samples (13).

The Moods and Feelings Questionnaire (MFQ) has been translated into Chinese and has demonstrated desirable validity and reliability in adolescent populations (14). Despite the potential benefits of the SMFQ, its applicability in Chinese adolescents has not been adequately validated. Research is needed to confirm its reliability and validity in this population to ensure effective use for early identification and intervention of depressive symptoms. In addition, existing studies have mainly used classical test theory (CTT) to analyze the psychometric properties of the SMFQ. Consequently, reliance solely on CTT may impede a nuanced and individualized understanding of the multifaceted construct being measured by the SMFQ.

The limitations of classical test theory (CTT) have been extensively discussed in the literature (15). One key limitation is that the psychometric properties of CTT are sample-dependent. This means that estimates of item difficulty (e.g., correctness rate), item discrimination (e.g., the correlation between an item and the total score), and reliability are all closely linked to the specific sample used (15). As a result, when the scale is applied to a different population, it must be re-normed.

CTT primarily focuses on total scores and assumes that all items contribute equally to the construct, failing to account for measurement error or the multidimensional nature of constructs (16). This fails to account for the different nature of the items and their performance variations across different populations (17). As a result, CTT does not fully reflect the different contributions of each item to the construct. As noted by several researchers (18–20), CTT’s inability to separate item-level variance and measurement error from true scores can obscure the more intricate characteristics of the construct.

To overcome the aforementioned limitations, opting for item response theory (IRT) analyses proves to be a more advantageous approach. Firstly, IRT analyses furnish insights into the validity of each scale, discerning effectively between respondents with varying underlying trait levels. Furthermore, these analyses shed light on the specific contribution of individual items to scale scores, providing a nuanced understanding of their significance in the overall measurement (21).

Moreover, IRT-based analyses extend their utility by identifying items that exhibit differential functioning among relevant groups, such as distinctions between boys and girls. In essence, the adoption of IRT provides a comprehensive and refined approach to understanding and addressing the complexities inherent in the psychometric properties of the SMFQ. For instance, studies have shown that IRT can uncover subtle biases in item responses, enabling more equitable and accurate assessments (22).

The Affective Self-Regulation (ASA) scale has been widely used in psychological research to assess individuals’ emotional states and their ability to regulate these emotions (23). Previous studies have demonstrated the reliability and validity of the ASA in various contexts, particularly in relation to mood disorders and emotional regulation (24, 25). Given its well-established psychometric properties, the ASA instrument serves as a robust tool for validating other psychological measures (26). In this study, we selected the ASA as the standard validity measure for the SMFQ-9, as it offers a comprehensive assessment of emotional regulation and motivational factors that are highly relevant to depressive symptoms (27).

The purpose of this study is to analyze the psychometric properties of the Short Mood and Feelings Questionnaire (SMFQ) among Chinese adolescents using item response theory (IRT). This study aims to provide a more nuanced and comprehensive evaluation of the SMFQ, offering insights into item-specific contributions, measurement accuracy across different trait levels, and differential item functioning among demographic groups. This approach seeks to enhance the utility of the SMFQ for rapid and reliable screening of depressive symptoms in large adolescent populations.

## Methods

### Participants

A total of 965 questionnaires were collected from several secondary schools in China. Before the formal analysis, we cleaned the questionnaires, and the screening criteria were that participants were considered invalid if they had any one missing answer. Using this criterion, we removed 59 invalid questionnaires and retained 906 valid questionnaires (93.4% valid responses). The mean age of the valid sample was 15.39, including 380 boys (42%) and 526 girls (58%).

### Measures

The SMFQ is a condensed 13-item version of the original 33-item MFQ. The questionnaire was developed in response to the need for a concise depression assessment tool that aimed to reduce the burden on participants while maintaining criterion validity (7). The questionnaire assesses depressive symptoms experienced in the past two weeks and is scored on a 3-point Likert scale (0 = not true, 1 = sometimes, and 2 = true) with a total score ranging from 0 to 26. Higher scores on the scale indicate more severe depressive tendencies. A substantial body of empirical evidence, including the original development study (7) and multiple cross-cultural validation studies (12, 28–30), supports the unidimensional structure of the SMFQ, which serves as a concise measure of overall depressive symptomatology in children and adolescents. For the item content of the SMFQ, please refer to **Tables 1** or **2**.

**Table 1:** Item fit statistics.

Item	*S-X* ^2^	*p*
1. I felt miserable or unhappy.	119.166	0.000
2. I didn’t enjoy anything at all.	25.240	0.561
3. I felt so tired I just sat around and did nothing.	26.500	0.599
4. I felt I was not worth much as a person.	53.671	0.002
5. I felt lonely.	24.245	0.669
6. I thought I wasn’t as good as other kids.	20.351	0.928
7. I was unhappy.	35.182	0.165
8. I didn’t get along with other people.	20.211	0.508
9. I felt I was a bad person.	35.138	0.086
10. I cried a lot.	19.776	0.802
11. I felt I was no good at all.	24.324	0.664
12. I felt I was being bad.	32.962	0.237
13. I felt just as good as other people.	34.424	0.224

**Table 2:** Item parameters.

Item	*a*	*b*1	*b*2
1. I felt miserable or unhappy.	1.050	-0.348	3.467
2. I didn’t enjoy anything at all.	1.790	0.128	2.448
3. I felt so tired I just sat around and did nothing.	1.763	-0.013	1.650
4. I felt I was not worth much as a person.	2.070	-0.101	1.575
5. I felt lonely.	1.813	-0.088	1.410
6. I thought I wasn’t as good as other kids.	1.867	0.241	1.591
7. I was unhappy.	1.863	-0.343	1.417
8. I didn’t get along with other people.	3.035	0.799	1.956
9. I felt I was a bad person.	2.916	0.536	1.810
10. I cried a lot.	2.147	0.059	1.608
11. I felt I was no good at all.	2.301	0.419	1.546
12. I felt I was being bad.	2.039	0.108	1.501
13. I felt just as good as other people.	2.178	0.425	1.699

The anhedonia scale for adolescents (ASA) (31) is used to assess adolescents’ loss of interest and pleasure in previously enjoyable experiences. Anhedonia, the loss of interest and pleasure in previously enjoyable experiences, is a core symptom of depression and a feature of other mental health and physical health problems. The ASA consists of 14 items on a 4-point Likert scale (0=never, 1=sometimes, 2=often, 3=always). The ASA scale comprises three dimensions. Dimension 1 consists of seven negatively worded items assessing enjoyment, excitement, and emotional flatness. Dimension 2 includes three positively worded items measuring enthusiasm, a sense of connection, and goal orientation. Dimension 3 comprises four negatively worded items evaluating effort, motivation, and inner drive. The ASA assesses the participant’s interest and pleasure in life over the past two weeks. Higher scores indicate a greater extent of disenchantment. The item content of the ASA questionnaire and their corresponding dimensions are provided in **Table 3**.

**Table 3:** ASA scale: items and corresponding dimensions.

Item Number	Item Content	Dimension
1	I enjoy doing things.	Dimension1
2	I feel excited when I try new things.	Dimension1
3	I often feel emotionally flat.	Dimension1
4	I feel enthusiastic about my goals.	Dimension2
5	I feel a strong connection to the people around me.	Dimension2
6	I am highly goal-oriented.	Dimension2
7	I work hard to achieve my goals.	Dimension3
8	I feel motivated to succeed.	Dimension3
9	I am driven to perform well.	Dimension3

The selection of the ASA as the criterion measure was driven by the objective of centering on anhedonia, a fundamental symptom of depression that is both highly pertinent and widely recognized in clinical practice. Anhedonia, defined as the absence of interest or pleasure in activities that were previously engaging, is widely regarded as a hallmark of depressive states. It plays a pivotal role in comprehending the emotional and motivational disturbances that characterize depression. Despite the fact that the ASA only measures a specific dimension of depression, its capacity to assess this fundamental aspect of depressive symptoms renders it a pertinent and valuable instrument for validating the SMFQ-9. Furthermore, the emphasis placed on anhedonia by the ASA is consistent with the overarching objective of evaluating depressive symptoms, particularly in adolescents, where disturbances in motivation and emotion are frequently observed.

### Statistical analysis

IRT analyses were performed using the mirt package (32) in the R software, using Graded Response Model (GRM) (33) according to the SMFQ response format. We chose the Graded Response Model (GRM) because it is well-suited for analyzing data from Likert-type scales like the SMFQ. Likert scales have ordered categories (e.g., “strongly agree” to “strongly disagree”), and the GRM is designed to handle such ordinal data. This model helps us understand how responses relate to underlying depressive symptoms by estimating two key parameters: item discrimination (how well an item differentiates between different levels of depression) and threshold parameters (the cut-off points between response categories).

Prior to formal use of GRM analyses, the data need to be tested for compliance with the key assumptions of the model, including unidimensionality, local independence, and fit of the IRT model. We then utilized Item Response Theory (IRT) to analyze the psychometric characteristics of the items. This analysis included examining item parameters, item information functions, and differential item functioning.

### Unidimensionality

Unidimensionality was assessed using exploratory factor analyses (EFA) using the R package psych (34). For EFA, the criteria for evaluating unidimensionality are that the first extracted factor should explain more than 20% of the variance (35), and furthermore, the ratio of the variance explained by the first factor to that explained by the second should be at least 4 (36).

### Local independence

The probability of reporting a symptom on the questionnaire was strictly dependent on the severity of the participant’s MFQ; therefore, items were independent of each other depending on the severity of the MFQ. To test this local independence assumption, we used the “residuals” function of the “mirt” package (32). Using the residual correlation matrix, we calculated Cramer’s V effectors for each item pair. We labelled item pairs as potentially locally dependent when the corresponding coefficients were above 0.20 (36).

### Item fit

Item fit is used to assess the fit of the IRT model at the individual item level. Item fit was examined using the *S-X* ^2^ statistic (37). This statistic compares the observed and expected response frequencies under the IRT model used and quantifies the difference between these frequencies. Items with *p* < 0.05 for the *S-X* ^2^ statistic were considered to not fit the IRT model (36).

### The graded response model parameters

For item parameters in the GRM model, each item is described by a discrimination parameter (*a*) and threshold parameters (*b*). The discrimination parameter indicates how well an item differentiates between individuals with different levels of the latent trait (θ). A higher discrimination parameter means the item is more effective at distinguishing between individuals who are just above and just below a certain level of the trait. The threshold parameters of an item correspond to the theta (θ) level of the latent trait necessary to respond to the corresponding anchor. The test includes two threshold parameters *b*1 to *b*2 (the number of threshold parameters for an item is equal to the number of response categories minus one). These threshold parameters indicate the θ levels at which the probability of choosing a higher response category transitions.

### Item information functions

In Item Response Theory (IRT), the Item Information Function (IIF) quantifies the amount of information an item provides about the latent trait (θ) at different levels of that trait. The IIF reflects how well an item can discriminate between individuals at various points along the latent trait continuum, based on the item’s discrimination and difficulty parameters. An item’s quality can be judged by its IIF, with high-quality items exhibiting high and broad peaks, indicating they provide substantial and precise information across a wide range of the latent trait.

### Differential item functioning

Differential Item Functioning (DIF) refers to a situation where an item exhibits different measurement properties for different groups of respondents with the same underlying trait level (θ), indicating potential bias. Analyzing DIF is important because it helps ensure that test items are fair and valid across diverse groups. By identifying and addressing items with DIF, test developers can improve the equity and accuracy of the assessment, ensuring that the test measures the intended construct equally well for all examinees.

## Results

### Assumption check

The one-factor EFA explained 38.7% of the total variance. Furthermore, the ratio of the first eigenvalue to the second eigenvalue is 5.44, which is greater than 4. These results indicate that the data satisfy the unidimensionality assumption. Regarding local independence, the Cramer’s V values for all items are small, with a maximum value of only 0.09, which indicates that there is sufficient independence between items. We used the *S-X* ^2^ statistic to assess item fit, and **Table 3** summarizes the results of item fit. Items with *S-X* ^2^ statistics corresponding to *p*-values less than 0.05 were considered not to fit the IRT model. The reliability and validity of the test can be improved by removing such unfit items. As shown in **Table 1**, only item 1 has a *p*-value less than 0.05 and can be considered for deletion.

### The graded response model parameters

**Table 1** summarizes the item parameters estimated using the GRM model. A discrimination parameter greater than 0.65 is considered the minimum acceptable lower bound (38). Additionally, the threshold parameters should follow a monotonically increasing order, ensuring that as the latent trait (θ) increases, the likelihood of endorsing higher response categories also increases. This ordering is crucial for the validity of the model, as it reflects the logical progression of responses corresponding to increasing levels of the underlying trait being measured (33).

As shown in **Table 2**, the discrimination parameter *a* for all items is greater than 1, indicating that each item has excellent discriminative power. Additionally, the threshold parameters *b*1 and *b*2 exhibit a monotonically increasing relationship, which aligns with the expected pattern. This orderly progression confirms that as the latent trait (θ) increases, the probability of endorsing higher response categories also increases.

### Item information functions

**Figure 1:**
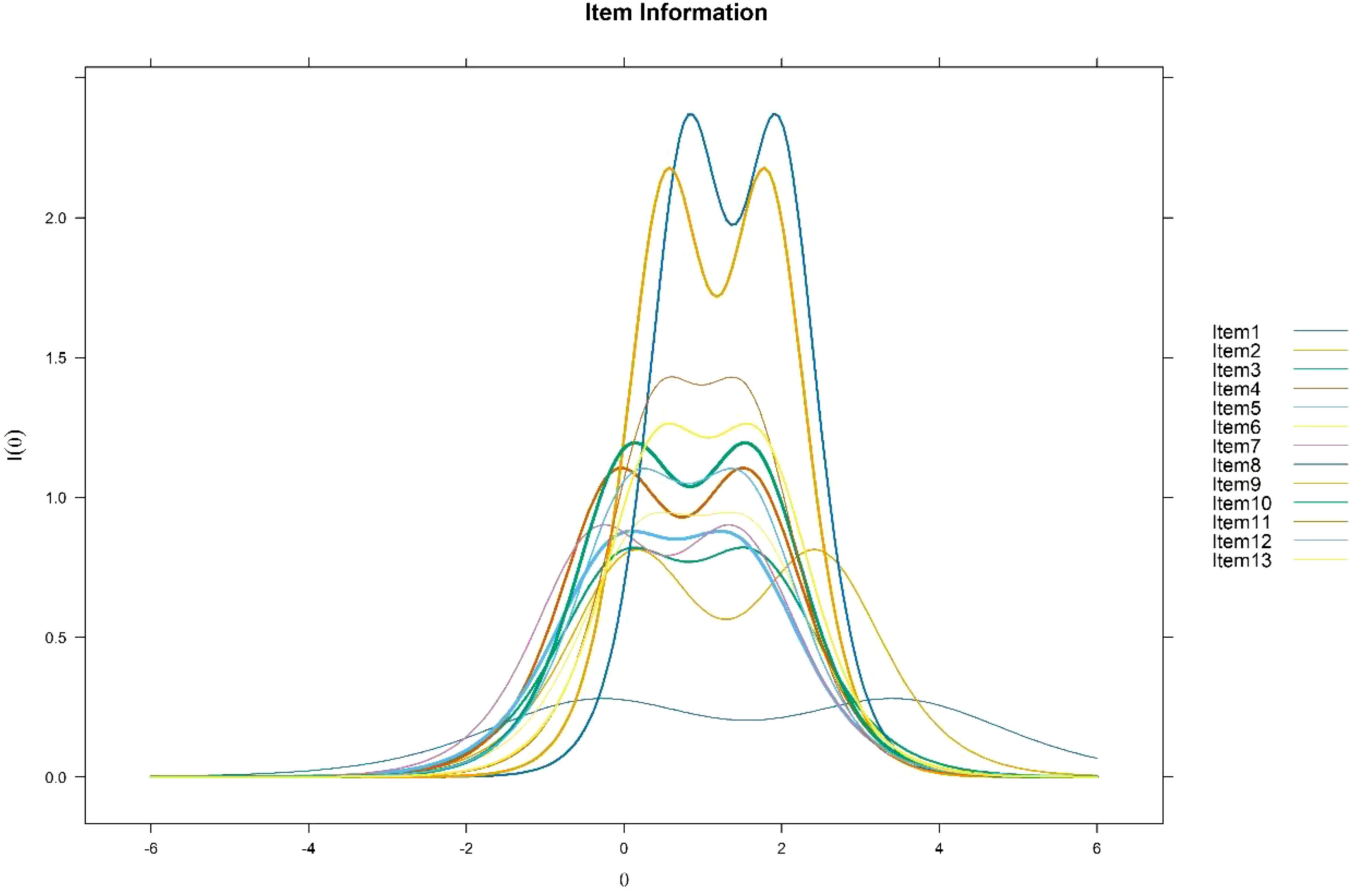
Item information functions.

**Figure 1** shows the information curves for all items. A flatter information curve indicates that the item provides less information, is less accurate, and contributes minimally to the overall test. Conversely, a steeper and higher information curve signifies that the item is more valid, more accurate, and contributes significantly to the measurement accuracy of the test. As can be seen in **Figure 1**, Item 1 has the bottom solid line with the flattest and least informative item information curve, which means that it provides the least amount of information. Therefore, Item 1 can be considered for deletion as its contribution to the effectiveness of the test is minimal.

**Figure 2:**
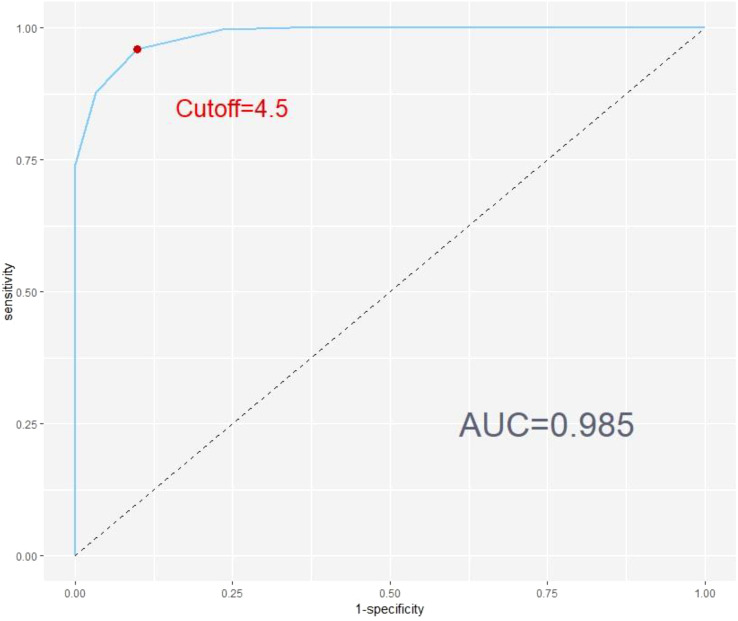
Receiver operating characte,ristic (ROC) curve for SMFQ-9 using full SMFQ diagnosis as criterion.

### Differential item functioning

We used the “DIF” function in the mirt package (32) of the R software to conduct a DIF analysis, employing the Wald statistic to test for the presence of DIF across different genders for both the discrimination and threshold parameters. If the *p*-value corresponding to the Wald statistic is less than 0.001, it indicates the presence of DIF in the item parameters. The existence of DIF suggests potential measurement bias, prompting us to consider deleting such items to ensure the fairness and accuracy of the assessment. **Table 4** summarizes the results of the DIF analysis for the differentiation and threshold parameters. From these results, it can be seen that the *p*-value for the difficulty parameter *b*2 for items 1, 3, and 6 is less than 0.001, indicating the presence of DIF. Therefore, to improve the accuracy and fairness of the test, these three items should be considered for deletion.

**Table 4:** Results of DIF analysis across gender.

Item	*p*-value		
	*a*	*b1*	*b*2
1. I felt miserable or unhappy.	0.705	0.000	0.277
2. I didn’t enjoy anything at all.	0.212	0.529	0.171
3. I felt so tired I just sat around and did nothing.	0.967	0.000	0.126
4. I felt I was not worth much as a person.	0.656	0.005	0.600
5. I felt lonely.	0.138	0.005	0.039
6. I thought I wasn’t as good as other kids.	0.711	0.000	0.064
7. I was unhappy.	0.746	0.726	0.887
8. I didn’t get along with other people.	0.515	0.032	0.770
9. I felt I was a bad person.	0.093	0.031	0.040
10. I cried a lot.	0.223	0.006	0.074
11. I felt I was no good at all.	0.272	0.007	0.392
12. I felt I was being bad.	0.307	0.009	0.135
13. I felt just as good as other people.	0.701	0.089	0.765

From a gender perspective, depressive symptoms may manifest in distinct ways, potentially leading to differential item functioning (DIF) across items. In this study, items 1, 3, and 6 exhibited gender differences, which could reflect variations in how depressive symptoms are expressed between boys and girls.

For item 1 (“I felt miserable or unhappy”), boys often express negative emotions through outward behaviors like anger or irritability, while girls are more likely to internalize these feelings as sadness or helplessness. This may lead boys to report fewer depressive feelings on this item. For item 3 (“I felt so tired I just sat around and did nothing”), girls may be more likely to report tiredness or low motivation directly, whereas boys might show these symptoms through behaviors like withdrawal rather than verbalizing fatigue. For item 6 (“I thought I wasn’t as good as other kids”), girls may be more affected by external judgments, such as appearance or social acceptance. Boys may also struggle with self-worth but are less likely to express it openly. As a result, girls tend to report more negative self-evaluations on this item.

In addition, the gender-related DIF observed in these entries provides potential direction for future revisions to the SMFQ-9. Specifically, more attention needs to be paid to the wording and content of the entries to ensure that they capture depressive symptoms in a manner that is valid for both genders. There is a need to consider revisions or additions to some of the entries to better reflect differences in the way boys and girls experience and express depressive symptoms.

### Item selection

To ensure the quality of the questionnaire, we meticulously screened the items based on several stringent criteria (1): The *p*-value of the item-fit *S-X* ^2^ statistic was less than 0.001 (2). The discrimination parameter (a) was greater than 0.65, and the threshold parameter (*b1*) was less than *b2* (3). The item information curves were excessively flat, indicating that the amount of information provided across all theta levels was minimal (4). The presence of Differential Item Functioning (DIF) in the item parameters.

Based on these criteria, our analysis yielded the following results. For the item-fit *S-X* ^2^ statistic, both item 1 and item 4 had *p*-values less than 0.05. However, all items met the required discrimination and difficulty parameters, so these items were not eliminated based solely on this criterion. When examining the item information curves, the curve for item 1 was nearly flat, suggesting insufficient information across all theta levels, thus warranting its removal. Based on DIF analysis, items 1, 3, and 6 displayed significant DIF, necessitating their elimination. Combining these findings, we improved the questionnaire by deleting items 1, 3, 4, and 6, resulting in a condensed version of the 9-item SMFQ that meets our measurement criteria, and in order to better differentiate it from the full version of the SMFQ, we abbreviate the condensed 9-item SMFQ as SMFQ-9.

The removal of Items 1, 3, 4, and 6 was based on thorough psychometric analysis, including item fit statistics, discrimination parameters, item information curves, and Differential Item Functioning (DIF). While these items were deleted, we ensured that the remaining items of the SMFQ-9 continue to adequately capture the core depressive symptoms, such as anhedonia (loss of interest or pleasure) and low mood. Specifically, Items 2, 5, and 7 continue to address key depressive symptoms, including persistent sadness, loss of interest, and lack of energy, which are central to the clinical understanding of depression.

Therefore, while the deletions were necessary to improve the psychometric properties of the instrument, the retained items maintain a comprehensive coverage of the core symptoms of depression, ensuring that the shortened SMFQ-9 remains a valid and reliable measure for depression screening.

Additionally, these four items may perform poorly due to cultural factors specific to the Chinese context. Item 1 (“I felt miserable or unhappy”): In Chinese culture, the expression of negative emotions is generally suppressed, and many people are unwilling to openly express sadness or unhappiness. As a result, this item received relatively flat responses. Item 3 (“I felt so tired I just sat around and did nothing”): Diligence and being busy are highly valued in Chinese culture, making it less likely for the feeling of being tired and doing nothing to elicit strong emotional responses. Item 4 (“I felt I was not worth much as a person”): In Chinese culture, self-deprecation or expressing feelings of low self-worth is less commonly expressed, particularly among adolescents who are often influenced by family and societal expectations. Item 6 (“I thought I wasn’t as good as other kids”): The Chinese education system emphasizes competition and family expectations, and adolescents may be less willing to openly express feelings of inferiority. Therefore, this item showed weaker responses.

### Validation of the revised SMFQ

We conducted an analysis of the Cronbach’s alpha reliability index for the revised SMFQ. The results indicated a highly satisfactory level of internal consistency, with a Cronbach’s alpha of 0.86. In addition, we used the marginal_rxx() function provided by the mirt package (32) to compute the test marginal reliability (0.824). This function calculates the test marginal reliability based on the parameters estimated by the IRT model, rather than directly using the total test score, thereby providing a more precise overall reliability assessment within the IRT framework. This suggests that the SMFQ-9 is a reliable measure for assessing the construct it is intended to evaluate.

Additionally, we compared the correlation coefficients of the full version of the SMFQ and the SMFQ-9 with the three subscales of the ASA, used as validity scales. It is worth noting that we adopted the approach proposed by Xiao et al. (39) and used the equated ability values (theta) to assess the correlation coefficients between the simplified SMFQ-9 and the full SMFQ. Additionally, we compared the correlations between both the simplified and full SMFQ and the three subscales of the ASA, which were used as validity measures.

The first subscale of the ASA is “enjoyment, excitement, and emotional flatness,” as in Question 2, “Nothing makes me feel excited.” Higher scores indicate more severe depression. The second subscale is “Enthusiasm, Connection, and Purpose” as reflected in item 8, “I feel enthusiastic”, with higher scores indicating a more positive outlook. The third subscale, “Effort, Motivation, and Drive” is shown by item 1, “I am not motivated to start doing things,” with higher scores indicating a more severe depressive mood. Therefore, the expected correlation between the three subscales of the ASA and the SMFQ is a positive correlation with subscale 1 and subscale 3 and a negative correlation with subscale 2.

The results of these correlations are summarized in **Table 5**. Both the full version and the SMFQ-9 show significant positive correlations with ASA subscales 1 (Enjoyment, Excitement, and Emotional Flatness) and 3 (Effort, Motivation, and Drive), and significant negative correlations with subscale 2 (Enthusiasm, Connection, and Purpose). These findings align with our expectations, supporting the notion that higher depression scores are associated with lower enthusiasm and connection, but higher levels of emotional flatness and drive-related issues.

**Table 5:** Criterion-related validity of the SMFQ-9.

Measures	ASA_Subscale 1	ASA_Subscale 2	ASA_Subscale 3	Full SMFQ
SMFQ-9	0.660^***^	-0.414^***^	0.568^***^	0.977^***^
Full SMFQ	0.657^***^	-0.407^***^	0.574^***^	–

Subscale 1, enjoyment, excitement, and emotional flatness; Subscale 2, enthusiasm, connection, and purpose; Subscale 3, effort, motivation, and drive; ^***^ *p* <.001.

Additionally, the validity scale correlations between the two versions of the SMFQ are very similar, demonstrating that there is little to no difference in their validity. The correlation between the two versions of the SMFQ is exceptionally high, at 0.977 (*p* <.001), indicating that the SMFQ-9 captures the same construct as the full version with remarkable accuracy.

To further explore the differences in scores on the simplified SMFQ-9 scale between different age groups, we divided the students based on their age distribution, using 15 years as the cutoff, as 15 years typically marks the transition from middle school to high school, with middle school students generally ranging from 12 to 15 years old. After categorizing the students into two age groups, we compared their scores on the SMFQ-9 scale. The results of an independent samples t-test indicated no significant difference between the two age groups’ scores (*t*=0.00, *p*=0.99), suggesting that there is no significant difference in depression levels between the two age groups.

### The optimal cutoff value for the SMFQ-9

In this study, the performance of the SMFQ-9 was evaluated using ROC curves. The critical value of the full SMFQ was used as a classification criterion, where a score of 7 or less indicated the absence of depression, and a score greater than 7 indicated the presence of depression. The results demonstrated that the area under the curve was 0.985, indicating an exceptionally high discriminatory ability of the SMFQ-9. The ROC curve is plotted in **Figure 2**.

From **Figure 2**, the optimal cutoff value for SMFQ-9 is 4.5. At the optimal threshold value of 4.5, the model’s sensitivity was 0.960, and its specificity was 0.902. This means that at this threshold, the model accurately identified 96% of the positive cases (actual positive samples) and correctly identified 90.2% of the negative cases (actual negative samples).

Overall, the results indicate that the SMFQ-9 performs exceptionally well in differentiating between the presence and absence of depression, making it a reliable tool for screening purposes. The high AUC value, combined with excellent sensitivity and specificity at the optimal threshold, underscores the model’s robustness in practical applications.

## Discussion

In this study, we conducted the first comprehensive assessment of the psychometric properties of the SMFQ for Chinese adolescents using item response theory (IRT). We used a graded response model (GRM) to conduct an in-depth analysis of the SMFQ’s item fit, item parameters, item information curves, and differential item functioning (DIF). The results showed that all items met the basic requirements for differentiation and threshold parameters. However, the item fit analyses showed that items 1 and 4 were poorly fitted, and the item information curve for item 1 was significantly flat, suggesting lower information value. In addition, items 1, 3, and 6 showed significant DIFs, suggesting possible bias in responses from different subgroups.

Based on these detailed psychometric evaluations, we streamlined the SMFQ to a more concise 9-item version by eliminating items 1, 3, 4, and 6. We then validated the reliability and validity of this simplified version, SMFQ-9. The Cronbach’s alpha for the SMFQ-9 was 0.86, indicating high reliability. To assess validity, we used the three subscales of the ASA as benchmarks and compared the validity correlations of the full SMFQ with those of the SMFQ-9. The results demonstrated that the validity correlations for the SMFQ-9 were very similar to those of the full version SMFQ. Additionally, the correlation coefficient between the SMFQ-9 and full SMFQ was exceptionally high, at 0.975.

These findings suggest that the SMFQ-9 maintains the integrity and measurement of the original questionnaire, while improving efficiency and reducing the burden on respondents. The SMFQ-9 is more suitable for practical use in clinical and research settings, and provides a reliable tool for large-scale, rapid screening of mood and emotion in Chinese adolescents.

In addition, the SMFQ-9 demonstrated excellent accuracy in differentiating between depressed and non-depressed adolescents, with an area under the curve (AUC) of 0.985. This performance is significantly superior to that reported in previous validation studies of the translated versions. For instance, two studies reported AUCs of 0.72-0.73, indicating a moderate level of accuracy (10, 12). Other studies reported AUCs ranging from 0.84 to 0.87, signifying good accuracy (40–43).

Additionally, some studies have found AUC values ranging from 0.51 to 0.82 in different groups (44). These results highlight the superior discriminatory power of the SMFQ-9 compared to its translated counterparts.

## Limitations

Despite promising results, this study has several limitations. A potential limitation of this study is that the sample was exclusively drawn from Chinese secondary schools, which may limit the generalizability of the findings to other populations. It is recommended that future studies include participants from diverse geographic regions and varying socioeconomic backgrounds in order to enhance the external validity of the results.

Additionally, one limitation of this study is that validity testing relied solely on the Adolescent Anhedonia Scale (ASA). While the ASA is a relevant measure of anhedonia, future research could benefit from incorporating additional depression measures, such as the Children’s Depression Rating Scale–Revised (CDRS-R) and the Reynolds Adolescent Depression Scale–2nd Edition (RADS-2). Including these tools would further validate the SMFQ-9 and enhance its construct validity, offering a more comprehensive assessment of depressive symptoms and providing a more nuanced understanding of depression in adolescents.

The cross-sectional design does not account for changes in the SMFQ-9’s properties over time. Longitudinal studies are needed to assess its stability and consistency. The study also didn’t differentiate between age subgroups within adolescents. Future studies should examine the SMFQ-9’s performance across different adolescent ages.

This study only used the IRT method to analyze the SMFQ. However, previous research has employed various analytical methods (45, 46), such as Classical Test Theory (CTT), Item Response Theory (IRT), and Rasch Model Theory (RMT), which have been widely applied in cross-cultural questionnaire analysis. Future research could draw on these approaches, combining multiple psychometric methods to comprehensively analyze the reliability and validity of the SMFQ, thereby gaining a more thorough understanding of its applicability and effectiveness across different cultures and populations.

The revised SMFQ-9 in this study, when compared to the full version of the SMFQ, initially determined a cutoff score of 4.5. However, this cutoff may have certain limitations. Future research should consider comparing the SMFQ-9 with other depression screening tools to further refine its cutoff score and validate its accuracy through additional methods, such as clinical interviews.

While the results of this study demonstrate that the SMFQ-9 is highly effective in differentiating between the presence and absence of depression, with an optimal cutoff value of 4.5, it is important to recognize that this cutoff value was determined solely by comparing the simplified SMFQ-9 to the critical value of the full SMFQ.

Future research should consider combining the simplified SMFQ-9 with other depression screening tools, structured clinical interviews, or other recognized diagnostic tools to validate this threshold and further refine its cut-off value, while also verifying its accuracy through methods such as clinical interviews.

In subsequent research, a direct comparison of the SMFQ-9 with the PHQ-9, a scale developed based on DSM criteria for depression, would be a valuable addition. The PHQ-9, a widely recognized instrument, utilizes a 4.0-point cutoff score to indicate the presence of depression, with higher scores reflecting more severe symptoms. By comparing the SMFQ-9’s cutoff value with that of the PHQ-9, it is possible to further validate the SMFQ-9 and refine its threshold for detecting depression, ensuring better alignment with established diagnostic criteria.

## Conclusion

In conclusion, this study reassessed the psychometric properties of the Short Mood and Feeling Questionnaire (SMFQ) using item response theory (IRT). Nine items meeting rigorous criteria were retained, forming the SMFQ-9. The SMFQ-9 showed high reliability and validity, strongly correlating with the original SMFQ. The critical threshold was set at 4.5, with an ROC curve area of 0.985, indicating excellent diagnostic accuracy. These findings suggest the SMFQ-9 maintains the robustness of the original while enhancing efficiency and reducing respondent burden, making it a reliable tool for assessing mood in adolescents.

## Data Availability

Publicly available datasets were analyzed in this study. This data can be found here: https://osf.io/4wdh5.
